# Transcriptomic and Network Meta-Analysis of Frontotemporal Dementias

**DOI:** 10.3389/fnmol.2021.747798

**Published:** 2021-10-15

**Authors:** Virginie Bottero, Fahed Alrafati, Jose A. Santiago, Judith A. Potashkin

**Affiliations:** ^1^Center for Neurodegenerative Diseases and Therapeutics, Chicago Medical School, Discipline of Cellular and Molecular Pharmacology, Rosalind Franklin University of Medicine and Science, North Chicago, IL, United States; ^2^NeuroHub Analytics LLC., Chicago, IL, United States

**Keywords:** frontotemporal lobars degeneration, frontotemporal dementia, neurodegeneration, Wnt signaling, MAPK signaling, valproic acid

## Abstract

Frontotemporal lobar degeneration (FTLD), also known as frontotemporal dementia (FTD), results in a progressive decline in executive function, leading to behavioral changes, speech problems, and movement disorders. FTD is the second most common cause of young-onset dementia affecting approximately 50**–**60,000 Americans. FTD exists in familial and sporadic forms, with GRN progranulin and C9orf72 mutations being the most common causes. In this study, we compared the sporadic and familial transcriptome within the cerebellum, frontal cortex, hippocampus, and Brodmann’s area 8 of patients with FTD to determine genes and pathways involved in the disease process. Most dysregulated genes expression occurred in the frontal cortex and Brodmann’s area 8 for genetic and sporadic forms of FTD, respectively. A meta-analysis revealed 50 genes and 95 genes are dysregulated in at least three brain regions in patients with familial mutations and sporadic FTD patients, respectively. Familial FTD genes centered on the Wnt signaling pathway, whereas genes associated with the sporadic form of FTD centered on MAPK signaling. The results reveal the similarities and differences between sporadic and familial FTD. In addition, valproic acid and additional therapeutic agents may be beneficial in treating patients with FTD.

## Introduction

Frontotemporal Lobar Degeneration (FTLD), also known as frontotemporal dementia (FTD), is a spectrum of clinical syndromes characterized by the progressive neurodegeneration of the frontal and temporal lobes of the brain. Damage to these brain regions results in behavioral changes, deficits in language and executive functions (Bott et al., 2014; Bang et al., 2015; Coyle-Gilchrist et al., 2016; Olney et al., 2017). FTD is the third most common cause of dementia after Alzheimer’s disease and dementia with Lewy bodies in individuals 65 years and younger, with a prevalence of 15–22/100,000 individuals (Coyle-Gilchrist et al., 2016).

FTD is a complex disorder with a highly clinical, genetic, and pathological heterogeneity. FTD has three clinical subtypes (Bott et al., 2014; Olney et al., 2017; Santiago et al., 2020). The behavioral variant FTD (Bv-FTD) is the most prevalent, and patients have behavioral deficits and loss of social functioning (Bott et al., 2014). In addition, there are two pathological variants of FTD, one with tau positive inclusions (FTD-tau) and the more prevalent form that is tau and alpha-synuclein negative (FTD-U) (Chen-Plotkin et al., 2008; Prasad et al., 2019).

In the context of genetics, about 40% of FTD patients are familial, 15% of which have an autosomal dominant inheritance pattern. Mutations in over 20 genes have been reported in FTD patients. For instance, mutations in progranulin (GRN), microtubule-associated protein tau (MAPT), and chromosome 9 open reading frame 72 (C9ORF72) are the most common (Woollacott and Rohrer, 2016; Capozzo et al., 2017). Other mutations including fused in sarcoma gene (FUS), TAR-DNA binding protein (TARDBP), valosin-containing protein (VCP), charged multivesicular protein 2B (CHMP2B), TATA box binding protein (TBP), sequestosome 1 (SQSTM1), ubiquilin 2 (UBQLN2), and optineurin (OPTN) have been recognized in less than 5% of the cases (Greaves and Rohrer, 2019).

Despite the progress in defining clinical subtypes and identifying genetic factors, the molecular mechanisms underlying the pathogenesis of FTD remain poorly understood. Several transcriptomic studies have provided insights into some of the dysregulated molecular pathways in FTD. For example, transcriptomic and pathway analyses of the frontal cortex showed marked differences in biological pathways between FTD patients with GRN (+) and without GRN (–) progranulin mutations (Chen-Plotkin et al., 2008). Upregulated genes in FTD GRN (–) subjects associated with lipid metabolism and downregulated genes were involved in the MAPK signaling pathway. Furthermore, upregulated genes in FTD GRN (+) were associated with TGF beta signaling and cell communication, whereas downregulated genes were related to calcium signaling.

Transcriptomic and network biology approaches have been key in the identification of dysregulated pathways and potential therapeutic targets in neurodegenerative diseases (Santiago and Potashkin, 2014; Santiago et al., 2017). Previous work in network-based studies in FTD has unveiled novel disease mechanisms. For example, analysis of a weighted protein-protein interaction network in FTD identified DNA damage response and cell waste disposal as important mechanisms of disease (Ferrari et al., 2017). Similarly, gene co-expression network analysis revealed that FTD genes were enriched in DNA metabolism, transcriptional regulation, and DNA protection in FTD patients’ frontal and temporal cortices (Ferrari et al., 2016).

In this study, we compared the transcriptomic profiles from different brain regions, including the cerebellum, frontal cortex, hippocampus, and Brodmann’s area 8 from sporadic and familial FTD subjects to identify potential mechanisms of disease and therapeutic targets, particularly pathways that may shed light on the distinction between the familial and sporadic subtypes (Chen-Plotkin et al., 2008; Olney et al., 2017). We incorporated network, pathway, and transcription factor analyses as a means of identifying dysregulated genes in pathways within FTD as well as in the familial and sporadic subtypes.

## Materials and Methods

### Database Mining, Curation, and Meta-Analysis

The NCBI GEO database^1^ and ArrayExpress database^2^ were searched on June 2020 for studies in which transcriptomic data was available from FTD patients. Three arrays containing samples from patients’ brains (E-MTAB-6189, GSE13162 and E-MTAB-656) were identified. The microarrays were curated using the database BaseSpace Correlation Engine (BSCE, Illumina, Inc., San Diego, CA, United States). Unfortunately, the dataset E-MTAB-656 did not meet the quality standards required by the BSCE curation methods. The meta-analysis tool in BSCE used a normalized ranking approach, allowing the elimination of any potential biases introduced by the use of different array platforms or the sample size. Using BSCE for differential gene expression analysis and meta-analysis, negative values, if any, were replaced with the smallest positive number in the dataset. Genes whose mean normalized test and control intensities were both less than the 20th percentile of the combined normalized signal intensities were removed. The activity of the gene in each dataset and the number of datasets in which the gene is differentially expressed were used to determine the scoring and ranking of each gene. The analysis included only genes with an absolute fold-change of 1.2 or greater and a *p*-value of 0.05 or less. BSCE computes the overlapping *p*-values between different gene expression datasets using a “Running Fisher” algorithm. *Post hoc* analysis was not performed. Unfortunately, the demographic and clinical information about the study participants publicly available was limited. E-MTAB-6189 sample information only included the age and the post-mortem interval (PMI). The patient information from GSE13162 included the number of male and female patients. Age, sex, and PMI to autopsy were not significantly different between cases and controls. The controls used in GSE13162 were defined as neurologically normal controls and were sampled from the University of Pennsylvania Center for Neurodegenerative Disease Research Brain Bank. The FTD cases were reviewed by a board-certified neuropathologist. In general, the control samples used in these studies were defined as neurologically healthy. Information about drugs, BMI, and comorbidities were not available. The sample population is summarized in Table 1.

**TABLE 1:** Sample population.

	**Gender (Male/Female)**	**Median age (IQR)**	**PMI (IQR)**
**GSE13162**			
Control	7/4	67 (54–75)	7 (5–14.5)
Familial (GRN + mutation)	3/4	71 (67–77)	6 (5–7)
Sporadic (GRN– mutation)	4/6	64 (53–72)	7.5 (3–11)
**E-MTAB-6189**			
Control	NA	66.5 (63–74)	5 (3.5–6.5)
Familial	NA	67.5 (58–80)	10 (5–12)
Sporadic	NA	73.5 (65–77)	7.5 (5–13)

*IQR: Interquartile range (25th–75th percentile). PMI: Postmortem interval in hours. NA, Non-available.*

### Pathway Enrichment Analysis

Entrez gene identifiers from the genes identified in the differential gene expression analysis and meta-analyses were imported into NetworkAnalyst for network and pathway analyses (Zhou et al., 2019). The Kyoto Encyclopedia of Genes and Genome (KEGG) pathway database was used as annotation source (Kanehisa and Goto, 2000). The pathways are ranked according to the number of hits and lowest *p*-value, with the two factors demonstrating a linear relationship. A *p*-value of less than 0.05 was considered significant. *Post hoc* analysis was not performed.

### Gene-Transcription Factors Interaction Analysis

Gene-transcription factors interactome was performed in NetworkAnalyst. Transcription factor and gene target data were derived from the Encyclopedia of DNA Elements (ENCODE) ChIP-seq data, ChIP Enrichment Analysis (ChEA), or JASPAR database (Lachmann et al., 2010; Wang et al., 2013; Fornes et al., 2020). ENCODE uses the BETA Minus Algorithm in which only peak intensity signal < 500 and the predicted regulatory potential score < 1 is used. ChEA transcription factor targets database inferred from integrating literature curated Chip-X data. JASPAR is an open-access database of curated, non-redundant transcription factor-binding profiles. A Venn diagram analysis was performed with the transcription factors identified with each database. Transcription factors were ranked according to network topology measurements, including degree and betweenness centrality.

### Gene-miRNA Interaction Analysis

The gene-miRNA interactome was performed in NetworkAnalyst. The Gene-miRNA interactome was carried out from comprehensive experimentally validated miRNA-gene interaction data collected from TarBase v.8.0 and miRTarBase v.8.0 (Chou et al., 2016, 2018; Karagkouni et al., 2018). miRNAs were ranked according to network topology measurements such as degree and betweenness centrality. Venn diagram analysis was used to identify the shared and unique set of miRNAs between familial and sporadic FTD patients. Biological and functional analysis of miRNAs was performed using miRNet 2.0 (Chang et al., 2020). The pathways are ranked according to the number of hits and lowest *p*-value. A *p*-value of less than 0.05 was considered significant. *Post hoc* analysis was not performed. This network software is publicly available and can be accessed at https://www.mirnet.ca/miRNet/upload/MirUploadView.xhtml.

### Gene-Chemical Analysis

Protein-chemical associated analysis was performed in NetworkAnalyst, which uses the literature curated gene-chemical database Comparative Toxicogenomics, a genomic resource available to the public that is derived from genes and proteins of toxicologic significance to humans (Mattingly et al., 2003). Chemicals were ranked according to network topology measurements, degree, and betweenness centrality.

## Results

### Database Mining for Brain Transcriptomic Studies

**FIGURE 1:**
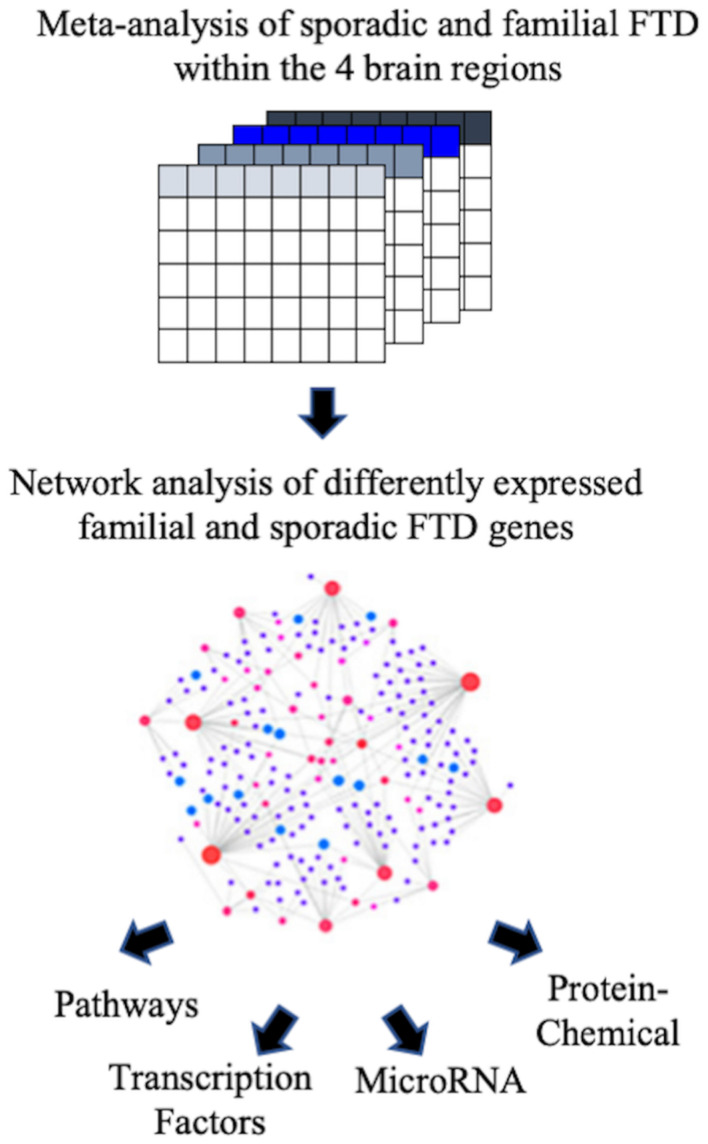
Flowchart of the study. The ArrayExpress and NCBI GEO databases were searched to identify studies that contained expression data from postmortem brain tissue of FTD patients. Microarray data from familial and sporadic FTD patients were curated, and meta-analysis was performed using the BaseSpace Correlation Engine (BSCE, Illumina, Inc., San Diego, CA, United States). The genes were then analyzed for functional pathways, transcription factors, miRNA, and chemical associations.

The Array Express and NCBI GEO databases were searched to identify studies that contained expression data from postmortem brain tissue of FTD patients and age-matched controls. Two independent studies that met our inclusion criteria were identified. Transcriptomic data obtained included sporadic and familial FTD data from four brain regions (frontal cortex, cerebellum, hippocampus, and Brodmann’s area 8). The description of the datasets analyzed in this study is presented in Table 2. The overall analysis strategy is shown in Figure 1.

**TABLE 2:** Datasets used in the study.

**Arrays**	**Platform**	**Conditions**	**Brain region**	**#of Patients (Familial | sporadic)**	**Gene features (Familial | sporadic)**
GSE13162	Affymetrix human genome U133A 2.0 Array	FTD patients with/without progranulin (GRN) mutations (i.e., familial vs. sporadic)	Frontal cortex	10 | 6	2921 | 5835
			Cerebellum	6 | 4	2739 | 1018
			Hippocampus	8 | 5	795 | 1543
E-MTAB-6189	A-GEOD-22844 Affymetrix human clarion D assay	Familial and sporadic FTD with C9ORF72 repeat expansion	Brodmann area 8	10 | 10	635 | 4171

### Analysis of Differentially Expressed Genes in Familial and Sporadic Frontotemporal Dementia Individuals

**FIGURE 2:**
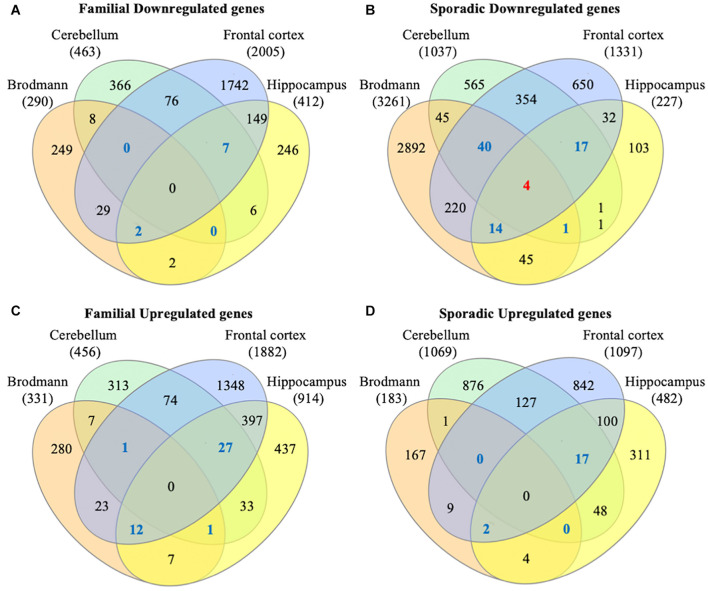
Venn diagram from FTD genes. Differentially expressed genes from familial and sporadic FTD patients were downloaded from curated databases. Venn diagram analysis showed that 50 genes were dysregulated in at least 3 out of the 4 arrays analyzed for familial FTD **(A,B)**, whereas 95 genes were dysregulated in at least 3 out of the 4 arrays for sporadic FTD **(C,D)**. Genes dysregulated in all 4 brain areas are shown in red, and those dysregulated in 3 out of 4 brain areas are shown in blue.

**FIGURE 3:**
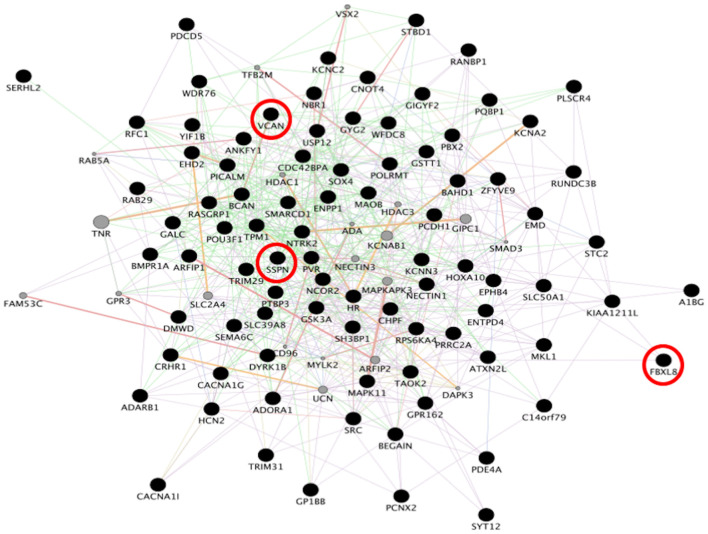
Network analysis from familial FTD genes. Network analysis of the 50 genes dysregulated in sporadic FTD was performed using GeneMANIA in Cytoscape v3.8.0. Input genes are shown in black circles. Purple, blue, and pink lines represent co-expression, co-localization, and physical interactions, respectively. FBXL8, VCAN, and SSPN were dysregulated in both familial and sporadic FTD.

**FIGURE 4:**
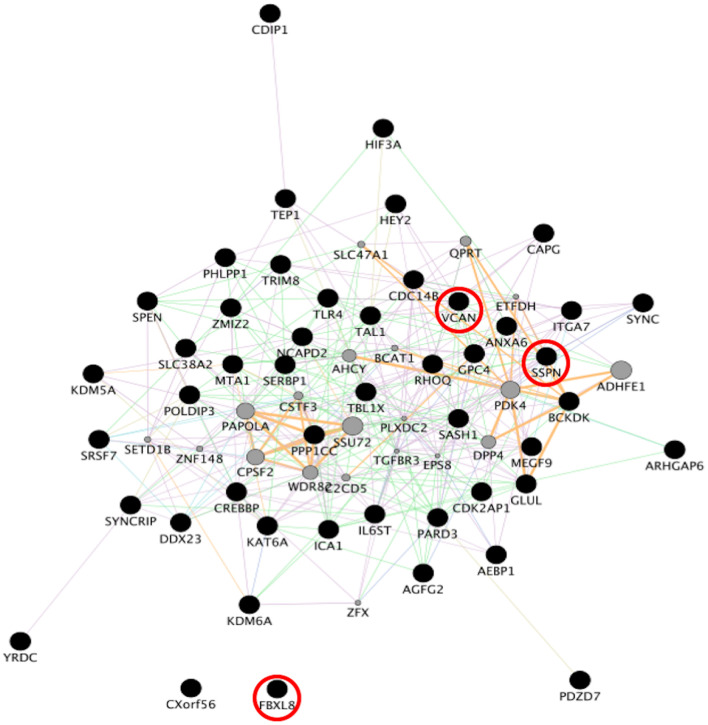
Network analysis from sporadic FTD genes. Network analysis of the set of 96 genes dysregulated in sporadic FTD was performed using GeneMANIA in Cytoscape v3.8.0. Input genes are shown in black circles. Purple, blue, and pink lines represent co-expression, co-localization, and physical interactions, respectively. FBXL8, VCAN, and SSPN were dysregulated in both familial and sporadic FTD.

To identify consensus among the different transcriptomic datasets from FTD patients, we performed a meta-analysis using BSCE (Supplementary Table 1). Meta-analysis of the four regions of familial or sporadic FTD microarrays was performed using only the set of dysregulated genes in the same direction with a fold-change of 1.2 or more (Figure 2). There were no shared genes between the four brain areas in the transcriptomic profiles from familial FTD subjects. Meta-analysis of the transcriptomic profiles from familial FTD identified 50 genes dysregulated in at least 3 out of 4 studies, including 9 downregulated genes and 41 upregulated genes (Figure 3 and Supplementary Table 2). Likewise, a meta-analysis of sporadic FTD transcriptomic profiles identified 4 genes downregulated in the four brain areas: protocadherin 1 (PCDH1), mitogen-activated protein kinase 11 (MAPK11), ectonucleoside triphosphate diphosphohydrolase 4 (ENTPD4), and programmed cell death 5 (PDCD5). In addition, 91 genes were dysregulated in 3 out of the 4 brain areas, including 72 downregulated genes and 19 upregulated genes (Figure 4 and Supplementary Table 2). Interestingly, 3 genes were shared between the familial and sporadic analysis: F-box and leucine-rich repeat protein 8 (FBXL8), versican (VCAN), and sarcospan (SSPN) (Figures 3, 4).

### Pathway Analysis of Familial and Sporadic Frontotemporal Dementia Genes

**FIGURE 5:**
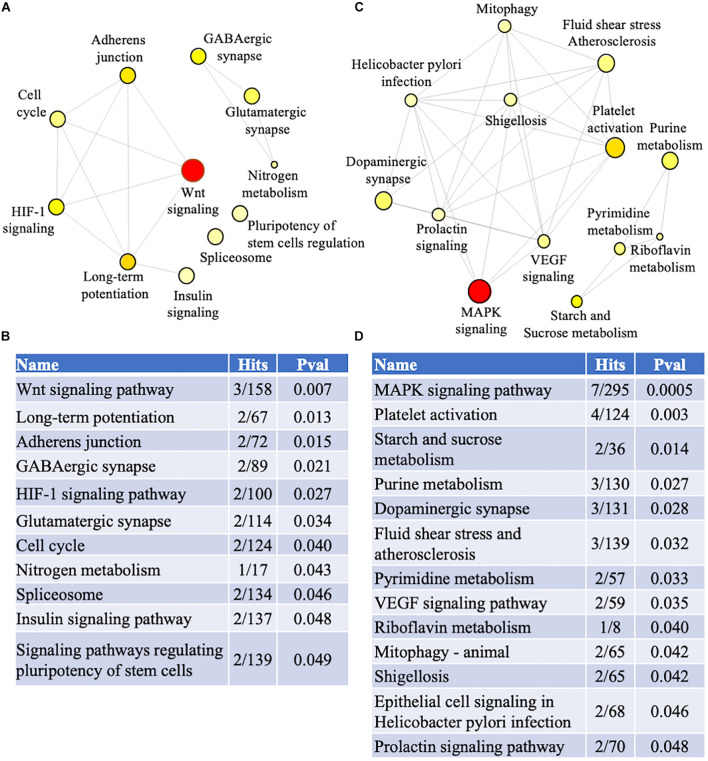
Pathway analysis. Genes identified in the meta-analysis from familial and sporadic FTD subjects were imported into NetworkAnalyst for pathway analysis **(A)** Network analysis of dysregulated pathways in familial FTD subjects. **(B)** Dysregulated pathways in familial FTD subjects. **(C)** Network analysis of dysregulated pathways in sporadic FTD subjects. **(D)** Dysregulated pathways in sporadic FTD subjects. The pathways are ranked according to the number of hits and lowest *p*-value, with the two factors demonstrating a linear relationship.

Biological and functional analysis of the familial and sporadic genes was performed using the KEGG database in NetworkAnalyst. Pathway analysis identified 11 and 13 dysregulated pathways in familial and sporadic FTD, respectively (Figure 5). All the pathways identified were unique to each FTD. Wnt signaling pathway was the most represented pathway in familial FTD with 3 genes associated (TBL1X, GPC4, and CREBBP). Sporadic FTD genes were predominantly associated with the MAPK signaling pathway. Seven sporadic FTD genes were linked to the MAPK signaling pathway (MAPK11, RASGRP1, CACNA1I, RPSKA4, TAOK2, and NTRK2).

### Gene-Transcription Factors Interaction Analysis

**FIGURE 6:**
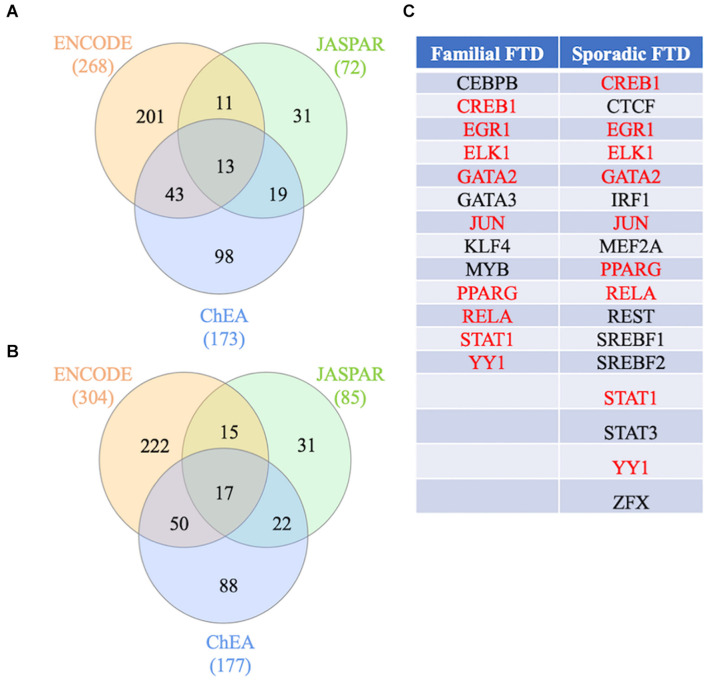
Transcription factors regulating FTD genes. **(A)** Transcription factor analysis from the familial FTD genes. **(B)** Transcription factor analysis from the sporadic FTD genes. Transcription factors and gene target data were derived from the ENCODE, ChEA, and JASPAR databases. Shared transcription factors between the 3 databases are shown in **(C)**. The transcription factors shared between familial and sporadic FTD are shown in red, and those unique to the specific FTD are shown in black.

To identify the main regulators of familial and sporadic FTD genes, transcription factor analysis was performed on NetworkAnalyst using three different databases (ENCODE, ChEA, and JASPAR). Venn diagram analysis revealed 13 shared transcription factors regulating the familial FTD genes (Figure 6A and Supplementary Table 3). Transcription factors CEBPB, GATA3, KLF4, and MYB were identified as unique master regulators of familial FTD genes. These transcription factors have been implicated in Alzheimer’s disease (Wang et al., 2019; Ndoja et al., 2020). To the best of our knowledge, these transcription factors have not been investigated in FTD models. Likewise, 17 transcription factors were identified as regulating the sporadic FTD genes (Figure 6B and Supplementary Table 3). In addition, 9 transcription factors were shared between familial and sporadic FTD datasets (Figure 6C). Interestingly, sporadic FTD genes were regulated by 8 unique transcription factors, including CTCF, IRF1, MEF2A, REST, SREBF1, SREBF2, STAT3, and ZFX. Among these factors, CTCF, MEF2A, and STAT3 have been associated with Alzheimer’s, Huntington’s, and Parkinson’s diseases (Gonzalez et al., 2007; De Souza et al., 2016; Wu et al., 2017). Transcription factor REST has been associated with the preservation of cognition in Alzheimer’s disease, dementia with Lewy bodies, and FTD (Lu et al., 2014).

### Gene-miRNA Interaction Analysis

**FIGURE 7:**
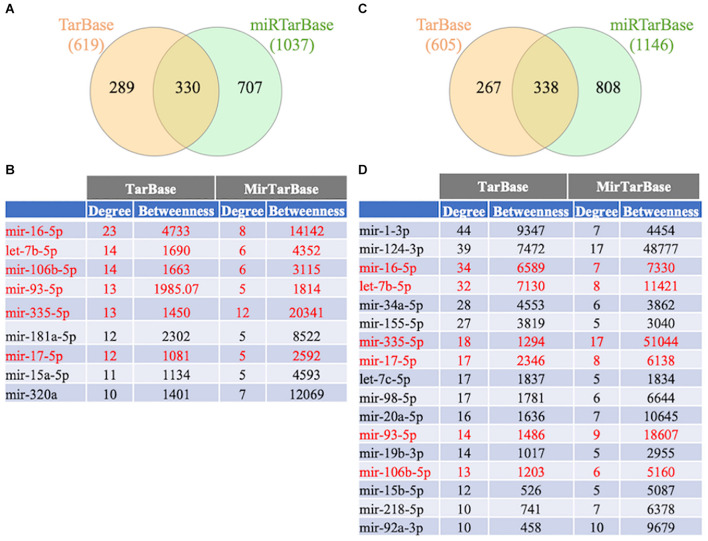
Venn diagram analysis of miRNAs. The FTD genes were uploaded to https://www.networkanalyst.ca/NetworkAnalyst/faces/home.xhtml. Gene-microRNA interactome was performed using TarBase v. 8.0 and miRTarBase 8.0. The top miRNAs were determined using a miRTarBase cut-off degree of 5 and TarBase cut-off degree of 10. **(A)** Venn diagram analysis of miRNAs regulating familial FTD. **(B)** Network topology measurements for the top miRNAs regulating familial FTD genes. **(C)** Venn diagram analysis of miRNAs regulating sporadic FTD genes. The miRNAs shared between familial and sporadic FTD are shown in red. **(D)** Network topology measurements for the top miRNAs regulating sporadic FTD genes.

To further study the regulation of the FTD genes’ expression, a gene-miRNA interaction network analysis was performed in NetworkAnalyst using 2 different databases (TarBase v.8.0. and miRtarBase v.8.0.). Venn diagram analysis showed that 330 and 338 miRNAs were shared between the databases that potentially regulate the familial and sporadic FTD genes, respectively (Figure 7 and Supplementary Table 4). In addition, 199 miRNAs were shared between the familial and sporadic FTD analyses (Supplementary Table 4).

To investigate the functional role of miRNAs in familial and sporadic FTD, we performed a pathway analysis using miRNet 2.0, a web-based platform for miRNAs functional analysis (Chang et al., 2020). Functional analysis of the top miRNAs regulating familial FTD genes associated predominantly with the immune system, angiogenesis, virus replication, and aging whereas miRNAs regulating sporadic FTD genes were enriched in pathways related to cell cycle, cell division, virus replication, cell death, hematopoiesis, T cell differentiation, and cell proliferation (Supplementary Table 5).

### Protein-Chemical Interaction Analysis

**FIGURE 8:**
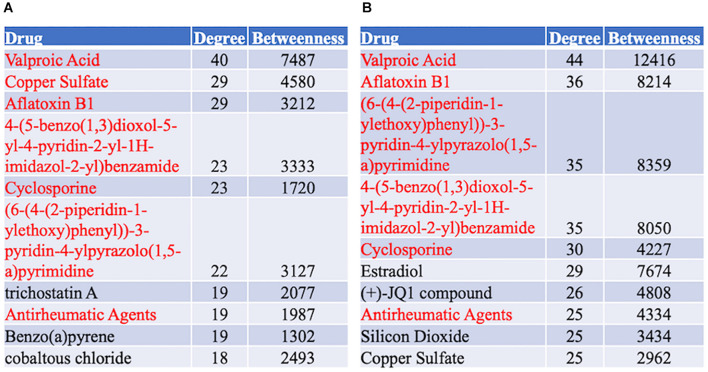
Top 10 Drugs associated with the FTD genes. **(A)** Drug analysis from the familial FTD genes. **(B)** Drug analysis from the sporadic FTD genes. The FTD genes were uploaded to https://www.networkanalyst.ca/NetworkAnalyst/faces/home. A Chemical-gene interactome was performed. Most of the chemicals were shared between familial and sporadic FTD analyses.

A protein–chemical interaction network analysis revealed drugs potentially helpful in treating familial and sporadic FTD. 370 and 504 chemicals were identified from familial and sporadic genes, respectively. The top 10 drugs associated with each FTD were identified in Figure 8. 223 chemicals were shared between the familial and sporadic FTD analyses (Supplementary Table 6).

## Discussion

### Meta-Analysis of Familial and Sporadic Frontotemporal Dementia Microarrays

A meta-analysis of the 4 brain regions of familial FTD microarrays identified 50 genes, 41 genes downregulated and 9 upregulated, in 3 out of the 4 studies. Annexin 6 (ANXA6) and adipocyte enhancer binding protein 1 (AEBP1) were the most significantly downregulated and upregulated genes, respectively, in familial FTD subjects compared to non-demented controls. Similarly, analysis of sporadic FTD identified 4 genes downregulated in the four brain areas: PCDH1, MAPK11, ENTPD4, and PDCD5. Although these genes have not been directly implicated in FTD, some have been associated with neurodegeneration. For example, ANXA6 interacts with tau and contributes to tau axonal localization (Gauthier-Kemper et al., 2018). AEBP1 is a transcriptional repressor involved in adipogenesis, macrophage cholesterol homeostasis, and activation of the NFKB pathway (Majdalawieh et al., 2020). Further, AEBP1 is associated with Braak staging and the degree of amyloid deposition, suggesting it is a marker of disease progression in Alzheimer’s disease (AD) (Shijo et al., 2018; Piras et al., 2019).

Interestingly, 3 genes were shared between the familial and sporadic FTD subjects, including FBXL8, VCAN, and SSPN. Genetic variants in VCAN are associated with cerebrovascular disease and dementia (Rutten-Jacobs et al., 2018). In addition, VCAN expression is dysregulated in ALS animal models (Mizuno et al., 2008; Forostyak et al., 2014). SSPN is differentially expressed in the blood of Alzheimer’s disease patients (Leandro et al., 2018). The involvement of these genes, however, in FTD is unknown and warrants further investigation.

### Biological and Functional Analysis of Familial and Sporadic Frontotemporal Dementia Genes

Biological and functional analysis of dysregulated genes in familial FTD identified several pathways, including WNT signaling, adherens junctions, GABAergic, HIF-1 signaling, glutamatergic synapse, spliceosome, and insulin signaling as the most overrepresented pathways. Familial FTD genes centered on the WNT signaling pathway. This finding reinforces previous investigations reporting a key role of WNT signaling in FTD. For instance, progranulin deficiency compromised neuronal cell survival by targeting WNT signaling *in vitro* and *in vivo* models of FTD (Rosen et al., 2011). Furthermore, the same study reported that upregulation of the WNT receptor Fzd2 promoted neuronal survival *in vitro* (Rosen et al., 2011). Collectively, these findings suggest that targeting WNT signaling is a potential therapeutic route for familial FTD.

Pathway analysis of sporadic FTD genes identified several pathways, including MAPK signaling, platelet activation, starch and sucrose metabolism, dopaminergic synapse, atherosclerosis, and VEGF signaling. Interestingly, sporadic FTD genes centered on MAPK signaling, the most overrepresented pathway. This is not surprising since MAPK signaling has been implicated in several neurodegenerative diseases, including FTD (Chen-Plotkin et al., 2008; Ahmed et al., 2020; Santiago et al., 2020). Of note, dysregulation of genes involved in the MAPK signaling has been reported in FTD subjects with progranulin mutations (Chen-Plotkin et al., 2008). In addition, genetic variants in VEGF conferred susceptibility to sporadic FTD in an Italian population (Borroni et al., 2008). However, there is no evidence of platelet activation, starch and sucrose metabolism, and atherosclerosis in FTD.

Although the association between the WNT and MAPK signaling pathways in familial and sporadic FTD, respectively, could be potentially interesting in the development of personalized treatments, these pathways are also involved in other neurodegenerative diseases. For example, these pathways have been implicated in the pathogenesis of amyotrophic lateral sclerosis (ALS), a fatal neurodegenerative disease that shares a clinical continuum and overlapping genetic factors with FTD. The WNT/β-catenin signaling pathway is responsible for the differentiation of neural stem cells into neurons as well as synaptic stability and plasticity (Jiang et al., 2021). Upregulation of receptors, co-receptors, and modulators of the WNT/β-catenin signaling pathway has been reported in several transgenic animal models and human spinal cord tissues from ALS patients (Yao et al., 1988; Gonzalez-Fernandez et al., 2019; Jiang et al., 2021). Most of the altered expression of WNT/β-catenin pathway components occur in astrocytes and motor neurons in ALS models (Yao et al., 1988). Interestingly, it is reported that the main drug to treat ALS patients, Riluzole, enhanced WNT/β-catenin signaling in neuronal cells (Biechele et al., 2010). Thus, targeting components of the WNT/β-catenin signaling pathway may be a possible therapeutic route for ALS and FTD. Similarly, dysregulation of MAPK signaling has been associated with ALS. MAPK members are the serine and threonine kinases that play a pivotal role in cellular proliferation, differentiation, apoptosis, and survival (Sahana and Zhang, 2021). Aggregates of abnormally phosphorylated MAPK components and TDP-43 have been found in motor neurons and spinal cord cells of ALS patients under stress conditions (Ayala et al., 2011). This aberrant hyperphosphorylation of MAPK members is associated with several pathophysiological defects involved in ALS including oxidative stress, neuroinflammation, and axonal transport disruption (Ayala et al., 2011). Several MAPK inhibitors have shown promise in various cellular and animal models of ALS and one inhibitor is currently being tested in clinical trials (Sahana and Zhang, 2021). The pathway convergence between ALS and FTD suggests that MAPK inhibitors might represent a potential therapeutic option for FTD patients.

### Transcription Factor Analysis

Transcription factor analysis revealed differences in the transcriptional regulation of familial and sporadic FTD genes. Analysis of familial and sporadic FTD genes identified 13 and 17 transcription factors, respectively. Four transcription factors, including CEBPB, GATA3, KLF4, and MYB were identified as unique master regulators of familial FTD genes. Among these transcription factors, the CCAAT enhancer-binding protein beta (CEBPB) and krupple like factor 4 (KLF4) have been documented previously in neurodegenerative diseases. For example, CEBPB regulates pro-inflammatory factors in microglia and is upregulated in AD (Wang et al., 2019; Ndoja et al., 2020).

Furthermore, CEBPB is also increased in the frontal cortex of HIV neurocognitive disorder patients and plays a role in regulating inflammation, metabolism, and autophagy in astroglia (Canchi et al., 2020). Although CEBPB has not been reported in FTD *per se*, the interplay between microglia and neuroinflammation is a central process in all neurodegenerative diseases. Likewise, KLF4 is involved in the microglial release of pro-inflammatory factors. Overexpression of KLF4 promoted the peptide Aβ42 induced neuroinflammation and neurotoxicity in the brains of transgenic AD model mice (Li et al., 2017). The involvement of these transcription factors in FTD warrants further investigations.

Transcription factor analysis identified 8 unique master regulators of sporadic FTD genes, including CTCF, IRF1, MEF2A, REST, SREBF1, SREBF2, STAT3, and ZFX. Several of these transcription factors have been implicated in neurodegenerative diseases. For instance, CTCF may play a role in regulating Huntington’s (HTT) promoter function in Huntington’s disease (De Souza et al., 2016). Moreover, the deletion of interferon regulatory factor 1 (IRF1) resulted in cognitive impairment in mice (Mogi et al., 2018). The myocyte enhancer factor 2A (MEF2A) promoted neuronal cell apoptosis *in vitro* and *in vivo* experimental models of Parkinson’s disease and some of its genetic variants associated with an increased risk of late-onset AD (Gonzalez et al., 2007; Wu et al., 2017). In addition, gene network analysis showed that Amyloid precursor protein (APP) is co-regulated by MEF2A (Wang et al., 2010). The repressor element 1 silencing transcription factor (REST) regulates a network of genes that mediates cell death and stress resistance in AD. Dysregulation of REST has been documented in AD, FTD, and dementia with Lewy bodies, and elevated levels of REST are associated with the preservation of cognition (Lu et al., 2014). Genetic variation in the sterol regulatory element-binding transcription factor 1 (SREBF1), known to play a role in lipid and cholesterol metabolism, is associated with increased susceptibility to dementia and AD (Spell et al., 2004; Carter, 2007; Reynolds et al., 2010). The signal transducer and activator of transcription 3 (STAT3), a major inducer of genes involved in apoptosis, cell growth, and inflammation, has been extensively implicated in neurodegenerative diseases. For example, STAT3 modulates glial activation and Aβ deposition leading to cognitive impairment in AD transgenic models (Toral-Rios et al., 2020). Moreover, inhibition of STAT3 rescued deficits in learning and memory in 5xFAD mice, suggesting it may be a potential therapeutic target for AD (Choi et al., 2020).

### miRNAs Regulating Familial and Sporadic Frontotemporal Dementia Genes

To investigate further the differences in regulation between familial and sporadic FTD genes, we performed a miRNA analysis. Functional analysis revealed that miRNAs regulating familial FTD genes were involved in the regulation of the immune system, angiogenesis, virus replication, and aging. In contrast, miRNAs regulating sporadic FTD genes were enriched in pathways related to cell cycle, cell division, virus replication, cell death, hematopoiesis, T cell differentiation, and cell proliferation. These findings, along with the pathways identified at the mRNA level, suggest that different regulatory mechanisms may be involved in the pathogenesis of familial and sporadic FTD patients.

### Chemical-Gene Interaction Analysis

Protein chemical network analysis identified several potential therapeutic agents for FTD. Valproic acid was identified as the highest-ranked chemical interacting with both sporadic and FTD genes. Indeed, some studies indicate that valproic acid is involved in WNT/β-catenin and MAPK signaling, pathways identified in this study as central in familial and sporadic FTD, respectively. For example, valproic acid stimulated neurogenesis in the adult hippocampus of transgenic AD mice through the activation of the WNT/β-catenin signaling pathway (Zeng et al., 2019). In addition, valproic acid promoted the differentiation of spiral ganglion neural stem cells and neurite outgrowth *via* the activation of the WNT/β-catenin signaling pathway (Moon et al., 2018). Furthermore, valproic acid elicited neuroprotective effects in the 1-methyl-4-phenylpyridinium (MPP^+^) model of PD in primary dopamine neurons via the activation of the MAPK pathway (Zhang et al., 2017). Further molecular studies in animal and cellular models of FTD will be key to understand the potential therapeutic value of valproic acid in FTD-associated neurodegeneration.

Interestingly, several recent studies have indicated that valproic acid may be a potential drug for neurodegenerative diseases. For instance, valproic acid treatment reduced the expression of pro-inflammatory cytokines and NF-κB signaling in PC12 cells treated with amyloid protein fragments suggesting that it may be neuroprotective via the attenuation of the inflammatory pathways (Zhao et al., 2018). Similarly, valproic acid treatment selectively reduced Aβ42 *in vitro* using 7PA2 cells transfected with human APP (Williams and Bate, 2018). Furthermore, valproic acid restored the physiological function of synapsin l, a synaptic protein important for neurotransmitter release in rat hippocampal neurons exposed to neurotoxic Aβ42 oligomers (Marsh et al., 2017). Recently, a bioinformatic analysis identified valproic acid as a potential drug for dementia (Potashkin et al., 2020). In the context of FTD, valproic acid improved neuropsychiatric symptoms including agitation, without exacerbating Parkinsonism in FTD subjects (Chow and Mendez, 2002).

Nevertheless, some studies have reported negative findings of the use of valproic acid for dementia. For example, an epidemiological study comprising 5,158 patients with bipolar disorder showed that valproic acid treatment increased the risk of dementia by 73–95% (Tsai et al., 2016). In addition, the use of valproic acid is associated with an increased risk of mortality in dementia patients (Kales et al., 2012). Therefore, more research studies into the potential mechanisms of valproic acid are needed to confirm its utility for dementia.

### Limitations and Future Research

Several caveats should be kept in mind when interpreting the results from this study. Although the curation methods of BSCE are rigorous, differences in microarray platforms and sample collection may bias the results. In addition, the demographic and clinical information about the study participants publicly available was limited. For example, information about drugs, comorbidities, and BMI was not available. Control samples were defined as neurologically healthy, but information about other diseases was not available. These confounding variables might have impacted the results of this study. In addition, FTD is a highly heterogeneous disorder that shares many genetic factors and pathological features with ALS. The results presented in this study are limited to sporadic and FTD with progranulin mutations or C9ORF72 repeat expansion. Therefore, future studies investigating the pathways dysregulated in FTD patients with other common mutations including MAPT, FUS, CHMP2B mutation and will be important to understand the underlying mechanisms in different genetic forms of FTD.

Finally, even though bioinformatic studies, in general, can lead to the identification of novel mechanisms of disease and therapeutics, findings should be confirmed in preclinical cellular and animal models.

## Conclusion

This study investigated the transcriptomic profiles from different brain regions from FTD subjects to identify pathways that may shed light on the distinction between the familial and sporadic FTD subtypes. We determined that most dysregulated gene expression occurred in the frontal cortex and Brodmann’s area 8 for genetic and sporadic forms of FTD, respectively. There were notable differences in the dysregulated pathways between familial and sporadic FTD subjects. We found that familial FTD genes were associated predominantly with the Wnt signaling pathway, whereas sporadic FTD genes were involved in the MAPK signaling. Top miRNAs regulating familial FTD genes associated predominantly with the immune system, angiogenesis, virus replication, and aging, whereas those regulating sporadic FTD genes were related to cell cycle, cell division, virus replication, cell death, hematopoiesis, T cell differentiation, and cell proliferation. In addition, valproic acid was identified as a potential treatment for FTD patients. Nonetheless, valproic acid has been suggested as a treatment for other neurodegenerative diseases, including AD and amyotrophic lateral sclerosis. Collectively, these findings suggest that different mechanisms may drive the disease process in familial and sporadic FTD patients. Further research on the transcriptional regulation and the molecular pathways involved in familial and sporadic FTD forms is warranted.

## Data Availability Statement

The original contributions presented in the study are included in the article/Supplementary Material, further inquiries can be directed to the corresponding author/s.

## Author Contributions

VB, FA, JS, and JP conceived and contributed to designs of the methods used in this study, analyzed the data, and wrote the manuscript. All authors have read and approved the final version of the manuscript.

## Conflict of Interest

JS was employed by NeuroHub Analytics, LLC. The remaining authors declare that the research was conducted in the absence of any commercial or financial relationships that could be construed as a potential conflict of interest.

## Publisher’s Note

All claims expressed in this article are solely those of the authors and do not necessarily represent those of their affiliated organizations, or those of the publisher, the editors and the reviewers. Any product that may be evaluated in this article, or claim that may be made by its manufacturer, is not guaranteed or endorsed by the publisher.

## Footnotes

1
https://www.ncbi.nlm.nih.gov/gds

2
https://www.ebi.ac.uk/arrayexpress/

## Supplementary Material

Supplementary Table 1 BSCE meta-analysis.

Supplementary Table 2 Pathway analysis.

Supplementary Table 3 Transcription factors analysis.

Supplementary Table 4 miRNA analysis.

Supplementary Table 5 miRNA pathway analysis.

Supplementary Table 6 Chemical analysis.
